# Similar neurocognitive outcomes after 48 weeks in HIV-1-infected participants randomized to continue tenofovir/emtricitabine + atazanavir/ritonavir or simplify to abacavir/lamivudine + atazanavir

**DOI:** 10.1007/s13365-018-0680-y

**Published:** 2018-10-08

**Authors:** Kevin Robertson, Paul Maruff, Lisa L. Ross, David Wohl, Catherine B Small, Howard Edelstein, Mark S. Shaefer

**Affiliations:** 1 AIDS Neurological Center, University of North Carolina at Chapel Hill, Chapel Hill, NC USA; 2 Cogstate Ltd., Melbourne, Australia; 3 ViiV Healthcare Company, 5 Moore Drive, Research Triangle Park, NC 27709 USA; 4 AIDS Clinical Trials Unit, University of North Carolina at Chapel Hill, Chapel Hill, NC USA; 5 New York Medical College, Valhalla, NY USA; 6 Weill Cornell Medical College, New York, NY USA; 7 Alameda County Medical Center, Oakland, CA USA

**Keywords:** HIV, HIV-associated neurocognitive disorder, Cogstate, Computerized battery, HIV-cognitive disorder

## Abstract

Human immunodeficiency virus (HIV)-associated neurocognitive disorders can persist in many patients despite achieving viral suppression while on antiretroviral therapy (ART). Neurocognitive function over 48 weeks was evaluated using a Cogstate test battery assessing psychomotor function, attention, learning, and working memory in 293 HIV-1-infected, ART-experienced, and virologically suppressed adults. The ASSURE study randomized participants 1:2 to remain on tenofovir/emtricitabine (TDF/FTC) and ritonavir-boosted atazanavir (ATV/r) or simplify to abacavir/lamivudine + atazanavir (ABC/3TC + ATV). Neurocognitive *z*-scores were computed using demographically adjusted normative data and were classified as “impaired” (defined as either a *z*-score ≤ − 2 or having 2 or more standardized individual test *z*-scores ≤ − 1); while higher scores (equaling better performance) were classified as “normal”. By *z*-scores, 54.7% of participants had impaired neurocognition at baseline and 50.2% at week 48. There were no significant differences (*p* < 0.05) in the baseline-adjusted performance between treatment groups for any individual test or by *z*-score. Specific demographic and medical risk factors were evaluated by univariate analysis for impact on neurocognitive performance. Factors with *p* < 0.10 were evaluated by backwards regression analysis to identify neurocognition-correlated factors after accounting for treatment, assessment, and baseline. Four risk factors at baseline for impaired neurocognition were initially identified: lower CD4 nadir lymphocyte counts, higher Framingham risk scores, and interleukin-6 levels, and a history of psychiatric disorder not otherwise specified, however none were found to moderate the effect of treatment on neurocognition. In this aviremic, treatment-experienced population, baseline-adjusted neurocognitive function remained stable and equivalent over 48 weeks with both TDF/FTC + ATV/r-treated and in the ART-simplified ABC/3TC + ATV treatment groups.

## Introduction

The use of ART in patients infected with HIV-1 has greatly increased survival and decreased the incidence of HIV-1 and AIDS-associated conditions, such as Kaposi’s sarcoma, *Pneumocystis jirovecii* pneumonia, and HIV-associated dementia. However, milder HIV-associated neurological disorders (HAND) have been noted in patients on ART, including those with suppressed viral loads. (Zhou and Saksena 2013; Simioni et al. 2010; Heaton et al. 2010; Saylor et al. 2016). Less is known about how the choice of ART impacts the likelihood of HAND in aviremic patients. Simplification studies provide a unique opportunity to investigate this issue, particularly given that current treatment guidelines, such as those from the US Department of Health and Human Services (DHHS) have endorsed regimen simplification for reducing pill burden, enhancing tolerability, improving quality of life, and decreasing the risk of some long-term toxicities (DHHS Panel on Antiretroviral Guidelines for Adults and Adolescents 2018). One simplification strategy is the discontinuation of low-dose ritonavir in aviremic patients, an approach that has been found to maintain virologic suppression, decrease the incidence of ritonavir-associated adverse events, and potentially reduce the incidence of CYP3A-mediated drug interactions (DHHS Panel on Antiretroviral Guidelines for Adults and Adolescents 2018; Elion et al. 2010; Gatell et al. 2007; Ghosn et al. 2010; Pavie et al. 2011; Santos et al. 2009; Sension et al. 2009; Soriano et al. 2008; Squires et al. 2010).

The randomized, open-label ASSURE (A Simplification Study of Unboosted Reyataz with Epzicom) study investigated the efficacy and safety of discontinuing ritonavir in virologically suppressed participants currently receiving a regimen of TDF/FTC plus ATV/r. Study participants were randomized to continue that regimen or to discontinue ritonavir while simultaneously switching to a fixed-dose combination (FDC) of the nucleoside reverse transcriptase inhibitors (NRTIs) abacavir and lamivudine ABC/3TC. The previously published 24- and 48-week efficacy results demonstrated that there was no significant difference between the two treatment groups by a time to loss of virologic response (TLOVR) analysis (Wohl et al. 2014; Wohl et al. 2016). To assist with understanding how the choice of ART may impact HAND in aviremic patients, one exploratory aim of the ASSURE study was to examine how simplification to ABC/3TC + ATV affected neurocognition through the 48 weeks of the study.

## Methods

### Clinical study description

Briefly, ASSURE (EPZ113734; NCT01102972) was a prospective, randomized, multicenter, open-label, and phase IV study that enrolled HIV-1-infected, ART-experienced adults (≥ 18 years of age) who had received a once-daily regimen of TDF/FTC 300 mg/200 mg (Gilead Sciences, Foster City, CA, USA) + ATV 300 mg (Bristol-Myers Squibb, Princeton, NJ, USA) boosted with ritonavir 100 mg (AbbVie, Chicago, IL, USA) for ≥6 months and had HIV-1 RNA ≤ 75 copies/mL for at least 28 days prior to the screening visit. As part of the inclusion and exclusion criteria, participants were excluded if they were HLA-B*5701-positive or had prior abacavir exposure, active CDC clinical category C disease, ongoing clinically relevant hepatitis and/or chronic hepatitis B infection (HBsAg+), or a creatinine clearance < 50 mL/min via the Cockroft-Gault method. Additional information regarding all inclusion/exclusion criteria, methods, and endpoints, including a link to the protocol, is available in the 24-week efficacy results publication (Wohl et al. 2014). All participants provided written informed consent to participate in the study, and the protocol was approved by the institutional review board for each study site.

After stratification by prior ART experience (TDF/FTC + ATV/r as the initial regimen or as the first/s switch regimen), eligible participants were randomized 2:1 to simplify their regimen to once-daily ABC/3TC 600 mg/300 mg (ViiV Healthcare, Research Triangle Park, NC, USA) plus ATV 600 mg or remain on TDF/FTC + ATV/r. The ASSURE study was powered to evaluate its primary endpoint, which was the proportion of participants with HIV-1 RNA < 50 copies/mL at week 24 by the time to loss of virologic failure (TLOVR) algorithm. Eighty-seven percent of participants in both treatment groups successfully maintained HIV-1 RNA < 50 copies/mL at week 24, demonstrating non-inferiority of simplification to ABC/3TC + ATV compared with continuation of TDF/FTC + ATV/r (Wohl et al. 2014). Results at week 48 were similar, with HIV-1 RNA < 50 copies/mL in 76% of participants taking ABC/3TC + ATV and 79% of participants taking TDF/FTC + ATV/r (Wohl et al. 2016).

### Neurocognitive assessments

Neurocognition was measured in the ASSURE study with a computerized cognitive test battery, the Cogstate Brief Battery (CBB; Cogstate; Melbourne, Australia). The CBB was selected because of its demonstrated sensitivity to HIV-related CNS impairment, (Maruff et al. 2009; Bloch et al. 2016) sensitivity to the effects of CNS active drugs generally (e.g., Grove et al. 2014; McIntrye et al. 2014), and to the effects of ART with CNS penetration (Winston et al. 2015). The CBB includes four cognitive tests which assess psychomotor function, attention, learning, and working memory which have previously been described in detail (Cysique et al. 2006). The CBB requires approximately 15 min for completion in HIV-infected adults; a single performance measure is generated for each of these four tests. Analyses of the neurocognitive data were conducted on the intent-to-treat exposed (ITT-E) population. All participants completed the entire CBB at a pre-baseline familiarization assessment, a baseline assessment and then an assessment at the week 24, week 48, and if an early withdrawal from the study occurred. Data from the familiarization visit was not used in the analyses.

To provide a measure of global neurocognition, a *z*-score was derived from the four tests in the CBB for each participant at each assessment. Each time, a study participant completed the CBB; their data were uploaded to a secure database and processed to provide the four primary outcome measures. Each outcome measure was then standardized using demographic-adjusted normative data and summed to compute a *z*-score, which was a neuropsychological composite of the four tests that was used to define global neurocognition. For each assessment, each participant’s neurocognition was categorized as either “impaired” (defined as either a *z*-score ≤ − 2 or having two or more standardized individual test *z*-scores ≤ − 1), while higher scores (equating to better performance) were classified as “normal.” The normative dataset used as a comparator in this analysis was obtained by Cogstate from a healthy, non-HIV-infected population. The normative dataset used in this analysis was stratified by age group, and was similar in composition to the ASSURE study participants in terms of age and gender distributions and where possible, was of similar ethnicity; as previously been described (Haddow et al. 2017).

### Statistical analysis

The primary exploratory analysis objective was to determine the extent to which 24 and 48 weeks of treatment with ATV + ABC/3TC versus ATV/r + TDF/FTC was associated with changes in neurocognition in the ITT-E population. Study participants must have attempted the baseline and either one or both of the week 24 and week 48 assessments to be included in analyses of neurocognition. Data were analyzed using a series of linear mixed models in which the baseline was modeled as a covariate and treatment group (ABC/3TC + ATV and TDF/FTC + ATV/r) and assessment were modeled as fixed factors. The *z*-scores, as well as the scores from each of the CBB tests, were modeled separately as the dependent variable. Any early withdrawal or unscheduled visit assessment done between week 12 and week 35 was reclassified into the week 24 assessment group; any assessment performed at week 36 or beyond was classified as week 48 assessments. Through week 48, results for the two treatment groups were compared using an analysis of covariance (ANCOVA) adjusted for baseline performance scores. Cohen’s *d* was calculated to estimate the magnitude of the differences between the two treatment groups; effect sizes of 0.2–0.4 are considered mild, 0.5–0.7 considered moderate, and ≥ 0.8 considered large (Cohen 1988). To validate the model assumption that the relationship between baseline and outcome were parallel for each treatment group, a baseline by treatment interaction was included in the model and removed only if the corresponding *p* value was > 0.05. A secondary analysis was also conducted to determine the extent to which 24 and 48 weeks of treatment with ATV + ABC/3TC versus ATV/r + TDF/FTC was associated with changes in neurocognition but restricted to the population of study participants with virologic suppression. The analysis population included participants with *z*-scores at the 24 or 48 week assessment visits and who had HIV-1 RNA < 50 copies/mL, as assessed by the time to loss of virologic response (TLOVR) algorithm, and the analysis compared the measures of cognition by treatment group and the effect sizes for treatment differences.

In another secondary analysis, the potential association between selected covariate risk factors (body mass index, CD4 nadir lymphocyte count, high-sensitivity C-reactive protein, depression, type I diabetes, type II diabetes, Framingham 10-year coronary heart disease risk score, interleukin 6 level, psychiatric disorders not otherwise specified, study stratification, the total weighted CNS penetration effectiveness (TWCPE) score), and neurocognitive performance (impaired versus normal) were examined using a two-tiered approach. First, a univariate analysis was conducted on each of the factors to determine their relationship to neurocognitive performance. Factors classified as different (i.e., *p* value < 0.10) were then submitted to a backwards regression process to identify those that correlated most strongly with neurocognition, after accounting for all the other relevant exploratory factors. The first stage, the univariate analyses, was conducted to identify the variables to be used in the backwards regression. This method was employed to avoid an over-saturated model at the start of the backwards regression process. The effect of the demographic and medical covariates on performance on the Cogstate battery was further investigated by determining which factors were associated with a change in performance over time for each Cogstate test. All data analyses were conducted using Statistical Analytic Software (SAS v9.2; Cary, North Carolina, United States).

## Results

Almost all (293/296; 99%) of the ASSURE study participants were included after having provided valid and complete baseline data for one or more of the four CBB tests. By the same criterion, 92% (272/296) of study participants completed an assessment at week 24 and 81% (240/96) at week 48.

All data received was submitted to test completion and data integrity checks (performed by Cogstate) to ensure that the data reflected performance on the test and that the demands of the test were understood. The analysis showed that the test battery was well tolerated in this study population; 99.67% of data were analyzed with ten test completion failures. After data cleaning, a total of 1089 tests were received across the pre-baseline, baseline, week 24, week 48, early withdrawal, and unscheduled visit assessments. The pre-specified data integrity checks were passed by 94.3% of the data at the analysis points (baseline, week 24, week 48, or withdrawal visits). Based on the high proportion of scores that met the integrity criteria, the entire data set was included in the ITT-E analysis.

### Study population

The enrolled participants were predominantly male and white (Table 1), although more than a third were African-American. All participants were from sites in the USA and Puerto Rico. More than 25% of participants identified as Hispanic or Latino. At baseline, nearly half of the participants had taken ≥ 1 ART regimen before starting TDF/FTC + ATV/r, and the median time on ART was approximately 3 years. The mean *z*-scores calculated at the baseline assessment for each of the two treatment groups were not significantly different, with a mean of − 1.155 (standard deviation 1.087; *n* = 180) for the ABC/3TC + ATV treatment group and a mean of − 1.1755 (standard deviation 1.136, *n* = 83) for the TDF/FTC + ATV/r treatment group.

**Table 1:** Baseline demographics and characteristics of study participants

	ABC/3TC + ATV (*N* = 199)	TDF/FTC + ATV/r (*N* = 97)
Median age, years (range)	44 (21–66)	42 (20–68)
Male, *n* (%)	155 (78)	79 (81)
Race, *n* (%)		
White/Caucasian/European heritage	122 (61)	55 (57)
African American/African heritage	65 (33)	37 (38)
Other	12 (6)	5 (5)
Hispanic or Latino ethnicity, *n* (%)	51 (26)	26 (27)
Median plasma HIV-1 RNA, log_10_ copies/mL	1.59	1.59
< 50 copies/mL, *n* (%)	192 (96)	93 (96)
Median CD4^+^ cell count, cells/mm (range)	492 (77–1196)	480 (108–1479)
< 200 cells/mm, *n* (%)	14 (7)	6 (6)
CDC Class C HIV infection, *n* (%)	37 (19)	17 (18)
Hepatitis C coinfection, *n* (%)	18 (9)	8 (8)
Median time on prior ART, days (range)	978 (177–4830)	1106 (199–7078)
≥ 1 prior regimen before starting TDF/FTC + ATV	43%	43%

*ABC/3TC*, abacavir/lamivudine; *ART*, antiretroviral therapy; *ATV*, atazanavir; *ATV/r*, atazanavir/ritonavir; *CDC*, Centers for Disease Control and Prevention; *TDF/FTC*, tenofovir/emtricitabine

At the start of the ASSURE study, 54.7% of the ART-experienced study participants were assessed as having impaired neurocognition **(**Table 2**)** and 50% or more of all participants were classified as having impaired neurocognition in the post-baseline assessments. While the overall proportions remained similar, the neurocognitive assessments for an individual participant did not always remain static. In an evaluation of the change from baseline (CFB) results at the week 24 assessment, approximately 25% of participant assessments had a change from their baseline classification status (from normal to impaired or from impaired to normal). Overall, a small proportion (13.3%) of participants went from a classification of impaired at baseline to normal at week 48, while a smaller proportion (8.7%) saw a change from normal to impaired neurocognition. Overall, at week 48, 50% of participants were classified as normal, while 50% were classified as impaired, similar to the proportions observed for the study population at baseline.

**Table 2:** Percentage of study participants classified as having either impaired or normal neurocognition at each assessment

Assessment	Classification	N*	% of total
Baseline	Normal neurocognition	120	45.3
Baseline	Impaired neurocognition	145	54.7
Week 24	Normal neurocognition	122	47.1
Week 24	Impaired neurocognition	137	52.9
Week 48	Normal neurocognition	120	49.8
Week 48	Impaired neurocognition	121	50.2
CFB to week 24	Change from normal to impaired neurocognition at week 24	29	11.2
CFB to week 24	Change from impaired to normal neurocognition at week 24	36	13.9
CFB to week 24	No change at week 24—impaired neurocognition	108	41.7
CFB to week 24	No change at week 24—normal neurocognition	86	33.2
CFB to week 48	Change from normal to impaired neurocognition at week 48	21	8.7
CFB to week 48	Change from impaired to normal neurocognition at week 48	32	13.3
CFB to week 48	No change at week 48—impaired neurocognition	100	41.5
CFB to week 48	No change at week 48—normal neurocognition	88	36.5

*CFB*, change from baseline

*This table count does not include 30 study participants who were missing a classification due to missing data on at least one of their assessments

The results for each of the individual tests and the *z*-scores were obtained for study participants with evaluable data and tabulated by treatment group (Table 3). There were no significant differences in the baseline-adjusted performance between the participants taking ABC/3TC + ATV and those taking TDF/FTC + ATV/r for any individual neurocognitive test or for the composite *z*-score. Consideration of the effect sizes indicated the magnitude for these group differences was uniformly small (i.e., *d* < 0.2).

**Table 3:** Overall comparison of ATV + ABC/3TC to ATV/r + TDF/FTC treatment groups at weeks 24 and 48 using the adjusted means from the linear mixed model analysis for each of the individual tests and the composite *z*-score for the intent to treat-exposed population

Test	Population		Week 48 adjusted mean difference (95% CI)	Cohen’s *d* effect size	*p* value
	ABC/3TC+ ATV, *n*	TDF/FTC+ ATV/r, *n*			
Week 24					
Detection^a^	172	82	0.004 (− 0.026, 0.033)	0.032	0.811
Identification^a^	172	83	0.015 (− 0.006, 0.036)	0.183	0.172
Once card learning^b^	175	84	− 0.006 (− 0.030, 0.018)	− 0.062	0.639
One back memory^a^	174	84	0.012 (− 0.012, 0.036)	0.134	0.316
*z*-score^b^	172	83	− 0.128 (− 0.39, 0.094)	− 0.152	0.257
Week 48					
Detection^a^	156	75	0.006 (− 0.026, 0.037)	0.050	0.721
Identification^a^	142	70	0.011 (− 0.010, 0.032)	0.147	0.291
Once card learning^b^	145	70	0.003 (− 0.027, 0.032)	0.024	0.865
One back memory^a^	144	70	− 0.001 (− 0.026, 0.023)	− 0.013	0.923
*z*-score^b^	142	70	− 0.054 (− 0.281, 0.172)	− 0.066	0.638

*ABC/3TC*, abacavir/lamivudine; *ATV*, atazanavir; *ATV/r*, atazanavir/ritonavir; *CI*, confidence interval; *TDF/FTC*, tenofovir/emtricitabine

^a^A negative difference score and negative effect size indicate that ATV + ABC/3TC performance was improved over ATV/r + TDF/FTC

^b^A positive difference score and positive effect size indicate that ATV + ABC/3TC performance was improved over ATV/r + TDF/FTC

As not all participants remained virologically suppressed through 48 weeks in either treatment group, a secondary analysis assessed whether there was any differential impact by treatment group on neurocognition, as assessed by the *z*-scores, for those participants who maintained virologic suppression (HIV-RNA < 50 copies/mL) through 24 or 48 weeks. By treatment group, for the comparison of ATV + ABC/3TC to ATV/r + TDF/FTC, there were 143 and 73 participants, respectively at week 24 who had remained virologically suppressed and had neurocognitive test results at baseline and week 24 and 142 and 70 virologically suppressed participants, respectively at week 48 with *z*-scores at baseline and week 48. The adjusted mean difference and 95% upper and lower CI for the comparison at weeks 24 and 48 were − 0.195 (− 0.435; 0.045) and 0.097 (− 0.332; 0.137), respectively. Cohen’s *d* effect size at weeks 24 and 48 were − 0.230 and − 0.120, and the *p* values were 0.111 and 0.414, respectively. Similar results were observed for analyses performed for each of the individual tests within the test battery and for all measures of neurocognition, the effect sizes for treatment differences remained small in magnitude and none were statistically significant.

### Covariate analyses

Analysis of the demographic and medical factors associated with a classification of impaired neurocognitive performance at the baseline assessment was performed to explore whether specific demographic or illness-related variables could moderate the effect of treatment. For the detection test, while the regression analyses identified high-sensitivity C-reactive protein as a covariate of interest, it no longer had any significance on the relationship between treatment and neurocognition after the backwards regression was conducted. For the identification test, the covariates psychiatric disorder not otherwise specified and CD4 nadir lymphocyte cell counts were found to be significant at the alpha level of 0.1 and remained in the model after baseline, treatment and visit were added. After adjusting for psychiatric disorder not otherwise specified and CD4 nadir lymphocyte count, the effect of treatment on neurocognition was similar (*p* value = 0.234 vs. *p* value = 0.178). For the one card-learning test, psychiatric disorder not otherwise specified, type II diabetes, stratification, and depression were significant after the univariate regression, however none of these covariates remained significant after the backwards regression process. For the one back test, depression was significant in the univariate analysis and remained in the model when treatment, baseline and visit were controlled. However, after adjusting for the effect of depression history on neurocognitive performance, the effect of treatment on neurocognition was similar (*p* value = 0.340 vs. *p* value = 0.571). None of the covariates were significant in the univariate analysis for the *z*-score (all *p* values > 0.1).

When performance on the neurocognitive tests at baseline was considered categorically as being impaired or normal (Table 4), differences were found for four factors: lower CD4 lymphocyte count at nadir, higher scores on the Framingham risk factor and higher values for interleukin 6, and having a history of psychiatric disorder not otherwise specified. For the continuous variables (Framingham risk, CD4 nadir lymphocyte count, and interleukin 6), the magnitude of differences in covariate score between impaired and normal neurocognition was expressed using Cohen’s *d*. For the categorical variable history of psychiatric disorder, the effect was expressed by computing the odds of impaired neurocognition with a history of psychiatric disorder compared to no medical condition. The magnitudes of differences in covariates for Framingham risk, CD4 nadir lymphocyte count, and interleukin 6 covariates were small (*d* < 0.2). However, a history of psychiatric disorder increased the odds of current classification of impaired neurocognition tenfold.

**Table 4:** Risk factors by participants classified as either normal or impaired neurocognition at the baseline assessment

Risk factor (category)	Neurocognitive classification	Mean^a^	Standard deviation	Mean difference	Magnitude of difference^b^	*p* value^c^
Body mass index	Normal	28.559	7.198			
Body mass index	Impaired	28.419	6.065	− 0.140	− 0.021	0.706
CD4 nadir	Normal	320.214	226.5			
CD4 nadir	Impaired	289.172	192.8	− 31.042	− 0.148	0.011
High-sensitivity C-reactive protein	Normal	3.957	9.735			
High-sensitivity C-reactive protein	Impaired	3.392	5.894	− 0.564	− 0.072	0.214
Depression (current)	Normal	0.094				0.191
Depression (past)	Normal	0.049				
Depression (not assessed)	Normal	0.004				
Depression (no medical condition)	Normal	0.306				
Depression (current)	Impaired	0.166			1.718 (0.96, 3.06)	
Depression (past)	Impaired	0.068			1.351 (0.62, 2.94)	
Depression (no medical condition)	Impaired	0.313				
Diabetes type II (current)	Normal	0.026				0.274
Diabetes type II (past)	Normal	0.008				
Diabetes type II (no medical condition)	Normal	0.419				
Diabetes type II (current)	Impaired	0.026			0.804 (0.27, 2.36)	
Diabetes type II (no medical condition)	Impaired	0.521				
Framingham score^d^	Normal	3.161	4.152			
Framingham score^d^	Impaired	3.757	5.525	0.597	0.120	0.030
Interleukin 6	Normal	2.452	3.350			
Interleukin 6	Impaired	3.517	5.748	1.065	0.222	0.000
Psychiatric disorders^e^ (current)	Normal	0.094				0.030
Psychiatric disorders^e^ (past)	Normal	0.004				
Psychiatric disorders^e^ (not assessed)	Normal	0.004				
Psychiatric disorders^e^ (no medical condition)	Normal	0.351				
Psychiatric disorders^e^ (current)	Impaired	0.102			0.948 (0.51, 1.75)	
Psychiatric disorders^e^ (past)	Impaired	0.045			10.528 (1.34, 82.51)	
Psychiatric disorders^e^ (no medical condition)	Impaired	0.400				
Stratification (initial antiretroviral regimen: ATV/r + TDF/FTC)	Normal	0.294				0.657
Stratification (initial antiretroviral regimen: NNRTI + TDF/FTC)	Normal	0.158				0.657
Stratification (initial antiretroviral regimen: ATV/r + TDF/FTC)	Impaired	0.370				
Stratification (initial antiretroviral regimen: NNRTI + TDF/FTC)	Impaired	0.177			1.123 (0.67, 1.87)	0.657
TWCPE^f^	Normal	6.129	0.693			
TWCPE^f^	Impaired	6.091	0.420	− 0.038	− 0.067	0.281

^a^Rather than mean, the proportion, where relevant, was calculated

^b^The magnitude of difference was calculated either by Cohen’s *d* or odds ratio for odds of impaired neurocognition; for the odds ratio, these were calculated as the odds of impaired neurocognitive performance with regard to a reference category. For depression, psychiatric disorders and diabetes type 2, the reference category is “no medical condition.” For stratification, the reference category is “Initial antiretroviral regimen: ATV/r + TDF/FTC.” Where there is not enough data in the relevant category, odds ratios are not calculated. The 95% confidence intervals for the odds ratios are also given

^c^*p* values were calculated by Student’s *t* test or by chi-squared for categorical variables (H0:no difference in means)

^d^10-year coronary heart disease Framingham risk score

^e^This variable encompassed all psychiatric disorders not otherwise specified

^f^Total weighted cerebrospinal fluid penetration effectiveness score

The effect of the demographic and medical covariates on performance for the CBB was further investigated by determining which factors were associated with change in performance over time for each Cogstate test. Any significant factors that were identified were added to a backwards regression model with that included terms for the baseline, assessment, and for treatment. Demographic and medical covariates were considered to have influenced performance if the relationship with change in performance remained statistically significant in the backwards regression model. These analyses identified no covariate that influenced a change in performance on the detection test, one card-learning test, or the *z*-score neurocognitive measurements. For the identification test, a history of psychiatric disorder not otherwise specified and CD4 nadir lymphocyte count was associated with cognitive performance after taking into account the baseline, assessment, and treatment effects. For the one back test, a history of depression was associated with cognitive performance after taking into account the baseline assessment as well as treatment effects. In both cases, the study treatments had no effect on neurocognition.

Taken together, these analyses indicate that after adjusting for baseline and relevant demographic and medical covariates there were no differences in performance between the ATV + ABC/3TC and ATV/r + TDF/FTC treatment arms for any of the Cogstate outcomes. While these risk factors were identified to be associated with neurocognitive performance over all assessments (baseline, week 24 or 48), none of these risk factors were found to moderate the effect of treatment on neurocognition.

## Discussion

The analysis of the assessment data indicated that the neurocognitive test battery was well tolerated in the USA/Puerto Rican study population and that performance on the battery was in accord with requirements, based on the very low rates of missing data and failure of data integrity across all study treatments and assessments. The estimates of within participant variability in performance were also consistent with those observed in previous studies of healthy adults performing the same Cogstate test battery. With levels of neurocognition taken into account, we observed no difference between the ATV + ABC/3TC and ATV/r + TDF/FTC treatment groups in psychomotor function, attention, working memory, or memory and also for a composite measure of these (*z*-score) which provided an index of general neurocognition. These results demonstrated that in this population, participants in the ATV + ABC/3TC treatment group were able to reduce their overall number of prescribed ART medications and simplify to an unboosted ART regimen with no negative impact on neurocognition. In all cases, the magnitude of the differences between treatment groups in baseline-adjusted group mean performance at the 48-week assessment were, by convention, trivial (i.e., effect sizes less than 0.2). Hence, when considered with the large sample size, these very small group differences suggest strongly the absence of any statistical significance or treatment terms in the analysis reflected that groups were truly not different, as opposed to arising from any low statistical power. Similarly, a secondary analysis evaluated the effect of the treatment simplification at the 24 and 48 week time points for those participants who had maintained good virologic suppression (HIV-RNA < 50 copies/mL). When the analysis was restricted to include only this virologically suppressed population and compared the two treatment groups, the conclusions from the intention to treat analysis remained unchanged. For all measures of cognition, the effect sizes for treatment differences remained small in magnitude and none were statistically significant.

Over half (54.7%) of this virologically suppressed (HIV-RNA < 75 copies/mL) study, population had impaired neurocognition at baseline. The reason for this increased rate of cognitive impairment is unclear and may reflect social or demographic characteristics of the sample itself, or some consequence of involvement in this study. While this classification enabled the comparison of the effects of the study treatments, the estimates of cognitive impairment observed in this study may not be reflective of cognitive impairment in the general population of HIV-infected, ART-experienced adults. The rates of impaired neurocognition reported for HIV-infected adults vary by study, and the rates observed in this analysis are consistent with some, but not all, studies. For example, one study enrolled 50 patients with HIV-1 RNA < 50 copies/mL and no self-identified neurocognitive impairment concerns at screening, however subsequent detailed neuropsychological testing with multiple assessments demonstrated that 32 (64%) had some level of neurocognitive dysfunction (Simioni et al. 2010). Another study in aviremic patients reported that depending on the assessment method used, 32 to 51% of their study population could be classified as having neurocognitive impairment (Wintson et al. 2010). Two additional studies (Cysique et al. 2011; Heaton et al. 2010) reported similar percentages of patients with impaired neurocognition using detailed neuropsychological testing, of 42% (49/116) and 52% (814/1555), respectively, although in these studies, not all participants were virologically suppressed (60% and 41%, respectively). Comparatively, lower rates of neurocognitive impairment (19%; 19/101) have been observed in an eight test version of the Cogstate battery in virologically suppressed patients (HIV-1 RNA < 50 copies/mL); the authors suggested that as the population under evaluation was a primarily older Caucasian population (median age 53 years; median time since HIV diagnosis 14 years), and could represent a “survivor” subgroup with advanced clinical presentation (Garvey et al. 2011).

Numerous studies have evaluated whether specific antiretroviral drugs or ART regimens, especially in patients with virologic suppression can impact neurocognitive function or what specific medical or demographic factors may impact neurocognitive function or the level of HIV-1 RNA in the CNS, but such studies have yielded mixed results (Arenas-Pinto et al. 2016; Cusini et al. 2013; Cysique et al. 2004; De Luca et al. 2002; Ellis et al. 2011; Giancola et al. 2006; Marra et al. 2009; Munoz-Moreno et al. 2008; Robertson et al. 2004, 2007, 2016; Smurzynski et al. 2011; Winston et al. 2010, 2013). In this ASSURE analysis, neurocognitive performance within treatment groups (TDF/FTC + ATV/r or ABC/3TC + ATV) remained stable over the 48 weeks of treatment and there was no significant difference between treatment groups in neurocognitive performance over the 48 week treatment period. While four risk factors at baseline were significantly associated with impaired neurocognitive performance on specific tests or when performance was assessed categorically, after adjusting for baseline and relevant demographic and medical covariates, there were no differences in performance between the ATV + ABC/3TC and ATV/r + TDF/FTC treatment groups for any of the Cogstate outcomes.

## Conclusions

In this aviremic, treatment-experienced population, there were no neurocognitive differences in the performance between the ATV + ABC/3TC and ATV/r + TDF/FTC treatment groups; the baseline-adjusted neurocognitive function remained stable and equivalent over 48 weeks with both treatment regimens.
